# “Doc' do I need an anterior cruciate ligament reconstruction? What happens if I do not reconstruct the cruciate ligament?”

**DOI:** 10.5704/MOJ.1503.008

**Published:** 2015-03

**Authors:** KS Dhillon

**Affiliations:** KPJ Selangor, Specialist Hospital, Shah Alam, Selangor, Malaysia

## Abstract

We are all aware that there has been a dramatic increase in the number of anterior cruciate ligament (ACL) reconstructions that are carried out here in Malaysia as well as around the world. The numbers of ACL injuries have undoubtedly increased over the years with greater participation of young adults in sporting activities. However it is not certain whether the increase in the numbers of reconstructions can be accounted for by the increasing numbers of ACL injuries. Without doubt commercial interests as well the influence of the biomedical companies have a role to play. In the past the rational for surgical treatment of an ACL tear was that the ACL is vital for knee function and that in the long term ACL deficiency will lead to more injuries of the meniscus and more degeneration of the joint. This belief was prevalent because the natural history of an ACL deficient knee was not known although the ultimate outcome of reconstruction of the ACL was not known either. However in past few years a substantial amount research has been published, which has elucidated the natural history of ACL deficient knees as well as the long term outcome of reconstruction of the ACL.

## Introduction

The Latin phrase ‘primum non nocere’, which is often wrongly attributed to Hippocrates, and which means ‘first do no harm’, has given rise to the principle precept of medical ethics that is taught to all medical students, called non-maleficence. It reminds us that we should consider the possible harm we may cause by medical intervention in the treatment of a patient. To prevent harm the concept of evidence- based medicine dictates that a critical enquiry is essential and reliance on clinical experience, textbooks and our local expert alone will not be sufficient.

Most of us will be familiar with the following scenario when Mr Smith walks into clinic after an injury to the knee. After a cursory examination of the knee the surgeon says,

‘Mr Smith you have an injury to the knee. I will send you for an MRI of the knee’. Looking at the MRI report the surgeon declares, ‘Mr Smith you have a tear of the anterior cruciate ligament (ACL) and you will need surgery to address this serious injury. You have a malalignment of the patella as well for which you need stabilization of the patella’.

The question is whether Mr Smith needs an ACL reconstruction? Needless to say that incidental MRI findings which do not and will not cause future disability, need no treatment.

### Prevalence of anterior cruciate injury

Anterior cruciate ligament (ACL) injury is a common sports injury with over two million such injuries occurring every year^1^. However the true prevalence of the incidence is not known due to lack of population based studies and the fact that some people with knee injuries do not seek treatment. Hospital based studies quote figures which vary from 30 to 81 per 100,000 people. A population based study of 56,659 people by Nordenvall et al showed an overall incidence of cruciate injuries of 78 per 100,000 people^2^.

### Diagnosis of injury to the ACL

Frequently most patients with knee injury are sent for magnetic resonance imaging (MRI). However an MRI is not necessary for the diagnosis of an injury to the ACL. Examination of the knee after acute knee trauma may not yield an accurate diagnosis due knee swelling and pain. However subsequent knee examination can give a relatively accurate diagnosis of an injury to the ACL. A positive pivot shift would rule in an ACL tear and a negative Lachman test would rule out a tear of the ACL. Lachman test is best overall for ruling in and ruling out an ACL tear while the anterior drawer test is inconclusive either way^3^. The Lachman test has an 85% sensitivity and a 95% specificity while an MRI of the knee has a 94% sensitivity and specificity in the diagnosis of a tear of the ACL^4^. Good history taking with a complete physical examination can provide a diagnostic accuracy of over 90%^5^. An MRI is useful for excluding other intra-articular injuries such as meniscal and chondral injuries. Hence an MRI has no additional value in diagnosis of ACL injuries when an examination shows anteriorposterior and rotational instability of the knee.

### Conservative or surgical treatment

The aim of treatment of a person with ACL tear would be to restore function, minimise symptoms, improve quality of life and minimise the risk of future complications^5^. It is commonly believed that a reconstruction of the ACL would fulfil all this aims. This belief is perpetuated by the fact that orthopaedic surgeons tend to over-estimate the results of reconstruction of the ACL in their patients. On an average they rate the outcome of the surgery in relation to knee function and activity level as significantly better (by 40 to 60%) than the patient does^5^.

Restoration of function, minimising symptoms and improving the quality of life are all inter-related. Hence we need to know what the symptoms are, what the patient cannot do and how the quality of life is affected. Level 3 scientific evidence shows that the activity level of the patient would be the most important factor that needs to be taken into consideration in decision making. The more active a person is in pivoting sports, the greater will the need for a reconstruction of the ACL to reach that level of activity^6^. There is level 1 scientific evidence that age is not a factor in making a decision to reconstruct the ACL^7^. However it is advisable to delay the surgery in children till the growth plates are almost closed4. Surgery is usually recommended for patients who are actively involved in pivoting sporting activities and those who have recurrent giving way of the knee during daily activities.

Does reconstruction of the ACL minimise the risk of future complication and improve quality of life Dunn *et al*^8^ in a study involving 6,576 active-duty army personnel showed that ACL reconstruction was protective against meniscal and cartilage injury. The study group had 3,795 subjects (58%) who had an ACL reconstruction and 2,781 subjects (42%) who were treated conservatively. Of those treated conservatively 32.6% underwent reoperations (meniscal, cartilage, ACL reconstruction surgery) as compared 12.7% reoperations (meniscal and chondral surgery) in the ACL reconstruction group. Subsequent ACL reconstruction was done in 26% of the patients who were initially treated conservatively. This retrospective follow up study as the authors admit has a selection bias. The authors were of the opinion that a randomised clinical trial to study the preventive benefits of ACL reconstruction would not be feasible ‘because of lack of equipoise’ but now they have been proven wrong.

Frobell *et al*^9^ have now published a five year outcome of a randomised trial for the treatment of acute anterior cruciate ligament tear. This level 1 scientific study compared the midterm (5years) patient reported and radiographic outcomes between those patients treated with rehabilitation plus early ACL reconstruction and those treated with rehabilitation and optional delayed ACL reconstruction. The cohort was 121 young active adults with a mean age of 26 years who had an acute ACL injury in a previously uninjured knee. All patients had similar structured rehabilitation and 62 patients were assigned to early ACL reconstruction while 59 were assigned to an option of having a delayed ACL reconstruction if the need arises. One patient was lost to follow-up at 5 years. They studied the 5 year outcome from baseline of the mean value of four out of five subscales of the Knee injury and Osteoarthritis Outcome Score (KOOS), the absolute KOOS (all five subscales), SF-36, Tegner activity scale, meniscal surgery, and radiographic osteoarthritis.

In the group of patients treated conservatively 51% of the patients needed a delayed ACL reconstruction. However all the outcome measurements were the same in the group treated conservatively and the group treated with ACL reconstruction. The meniscal surgeries rates, radiographic osteoarthritis and all functional scores were the same in both groups. The results were hence the same in patients treated conservatively, and those treated with early or delayed reconstruction of the ACL. The authors concluded that these results should encourage clinicians and young active adults to consider rehabilitation as the primary mode of treatment for an acute ACL tear. In other words about 50% of the patients will not need an ACL reconstruction if they are treated with structured rehabilitation. If we follow the prevailing advice that all young active patients should have reconstruction of the ACL^10^, about 50% would be having unnecessary surgery. This study however does not apply to professional athletes as well as to patients who are involved in less than moderate activity.

The outcome of the findings in this first ever level 1 study has not got everyone taking solace in the fact that 50% of the patient did not need a reconstruction of the ACL, since a commentary in the Journal of Bone and Joint Surgery^11^ suggest that the results are open to interpretation and that some may ask if 50% of the patients will need subsequent surgery, ‘why wait’. The logical answer would be that waiting will prevent unnecessary surgery and possible complications in 50% of the patients and not to mention the financial savings which would substantially be more now than the figure of $1 billon going by the year 2000 estimate in the US^10^.

The frequency of delayed reconstruction of ACL after initial conservative treatment in this study of young active individuals by Frobell *et al* is high compared to other studies^12^. Other studies have reported frequencies that range from 16% to 35% but these studies are not comparable because of the nature of the study, patient selection, criteria for surgery, the nature of surgery and treatment.

A reflection of the number of patients who will need a delayed reconstruction of the ACL in a general population is provided by a level 2 study by Neuman *et al*^13^. They followed up 100 consecutive patients with a complete tear of the ACL, which was confirmed by arthroscopy, for 15 years. All patients were treated conservatively with rehabilitation. The study excluded those who participated in professional sports and were unwilling to reduce their activity level. They found that 23% of the patients required a delayed reconstruction of the ACL between 6 months and 11 years. This would translate to 77% of patients not requiring an ACL reconstruction at 15 years follow-up.

### Return to sports

In the past reconstruction of ACL has been advocated as a requirement for return to competitive sports after a tear of the ACL. Ardern *et al*^14^ have done a systematic review and meta-analysis to determine post-operative return to sports outcomes after ACL reconstruction surgery. Their review found that although 90% 0f the patients achieved successful surgical outcome in terms of impairment based measurement of knee function and a 85% successful activity based outcome, only 44% of patients returned to competitive sport and approximately 63% of patients returned to pre-injury level of sports participation.

Swirtun *et al*^15^ in a study involving 46 patients aged between 18 and 50 years, with an acute unilateral ACL tear, where the treatment was self- selected by the patient to have conservative treatment or reconstruction of the ACL, found no difference in activity level at a 5.6 years follow up. In this study 24 patients had conservative treatment and 22 had an ACL reconstruction. In fact the conservative group had a significantly better outcome in the knee related Quality of Life (QOL) domain of the KOOS than the patients with ACL reconstruction.

The study by Frobell *et al*^9^ showed a modest return to preinjury activity level at 5 years after a tear of the ACL and there was no difference between the groups treated with early ACL reconstruction, delayed ACL reconstruction or those treated with rehabilitation alone.

### Secondary meniscus injury

Early ACL reconstruction is recommended to minimise the risk of meniscal tears. Church *et al*^16^ in a retrospective review of 183 patients compared the incidence of meniscal tears in patients who underwent ACL reconstruction early (within 12 months of injury) and those who underwent reconstruction late (after 12 months). They showed that the incidence of meniscal tear was higher in the late reconstruction group (71.2 % versus 41.7%). They recommended early surgery to prevent meniscal injury. This study was not a randomised study comparing early with late reconstruction and the numbers in each subgroup were small for statistical comparison.

A level 4 case series by Yoo *et al*^17^ also showed an increased likelihood of medial meniscus tear when ACL reconstruction was delayed. This study involved a highly selected group of patients. They selected 31 patients from among 311 patients who had concurrent meniscal repair and ACL reconstruction. The selection criteria was the availability of two MRI studies, one at the time of injury and another at a later date when the patient opted for a delayed ACL reconstruction. They showed that the incidence of medial meniscus injury in patients with chronic ACL deficiency increased from 55% at the first MRI studies to 84% at the second MRI studies. The mean between-study time was 36.8 months. Papastergiou *et al*^18^ in retrospective study of 451 patients showed that the prevalence of meniscal tears is significantly higher if ACL reconstruction is delayed for more than three months

These are retrospective observational studies with compromising interpretation of their findings^9^. The real frequency of secondary meniscal injury or meniscal surgery is not known. The first and only high quality randomised control trial done by Frobell *et al*^9^ showed that there was no statistically significant difference in the number of knees having meniscus surgery over a 5-year follow up after an ACL injury, between groups treated with rehabilitation, early ACL reconstruction or delayed reconstruction. Time to event analysis of proportion of meniscus treated with surgery also did not show any difference between the groups. In the past it was believed that reconstruction of the ACL reduces the risk of meniscal tears but this study did not show that reconstruction of the ACL reduces the risk of meniscal tears or not reconstructing the ligament increases the prevalence of meniscal tears.

### Secondary osteoarthritis

ACL deficiency and partial or total meniscectomy are well known risk factors for post-traumatic OA. Ajuied *et al*^19^ have recently reported the first meta-analysis on the development and progression of OA after an ACL injury at a minimum of 10 years follow-up using Kellgren-Lawrence (K-L) classification of radiographic OA. Their systemic review and meta-analysis showed a 20.3% prevalence of grade 3 and 4 OA in patients with ACL deficiency as compared to 4.9% in the contralateral ACL intact uninjured injured knee.

The study also showed that the relative risk (RR) of developing even minimal (grade 1 and 2) OA was 3.89 and the relative risk of moderate to severe (grade 3 and 4) OA was 3.84 after an ACL injury irrespective of whether the treatment was surgical or conservative. The nonoperatively treated knees had a higher relative risk of developing any grade of OA as compared to those knees which had reconstructive surgery. However the progression of OA to moderate and severe OA after 10 years was significantly higher in the reconstructed knees (RR 4.71).

Porat *et al*^20^ studied the prevalence of radiologic OA in a group 205 male league soccer players and found that, at 14 years after the initial ACL injury 78% of the injured knees had radiologic evidence of OA. Grade 2 or more K-L radiographic changes were seen in 41% of the injured knees and 4% of the uninjured knees. There was no difference in the prevalence of radiologic OA between knees treated conservatively or with reconstruction. The patient relevant outcome was affected with 80% of the subjects reporting a reduced level of activity after the injury. However the level of activity was the same in patients with and without OA. Fifty-five percent of the subjects reported participation in a level 5-6 activity (high level recreational activity) and 53% reported a level 2-4 activity participation (easy to moderate load at work). In fact 7.8% were still involved in organised soccer 14 years after the ACL injury. The study found no difference in the prevalence of OA or symptoms in subjects treated conservatively or surgically.

Although the meta-analysis by Ajuied *et al*^19^ showed a higher relative risk of OA in patients treated without reconstruction, the study by Porat *et al*^20^ in soccer players showed no difference in the prevalence of radiographic OA in those treated conservatively or with surgery. This could partly be due to the knee protective neuromuscular rehabilitation that soccer players go through before resuming sports as compared to others who are not involved in competitive sports.

Neuman *et al*^18^ showed in a prospective level 2 study involving 100 consecutive patients, who were treated by neuromuscular rehabilitation and activity modification after an ACL injury, that it is possible to achieve a good knee function with a low prevalence of post-traumatic OA at 15 years follow-up. In this study the attrition rate was low with 6 patients lost during follow-up. A delayed ACL reconstruction was necessary in 22 patients (23%) at between 6 months and 11 years. Tibiofemoral OA of K-L grade 2 or more was present in 16% of the patients and all these patients belonged to the group who had - meniscectomy. - None of the patients with an intact meniscus had OA. The OA occurred in the same compartment as the Meniscectomy. Thirty-five percent of the patients with ACL reconstruction had tibiofemoral OA whereas 11.2% of the patients without reconstruction had tibiofemoral OA.

As far as symptoms were concerned 67% of the patients were asymptomatic at 15 years. Of these 67%, OA was absent in 59% and present in 8%. Thirty-three percent of the patients were symptomatic, of whom 24% had no OA and 8% had OA. Patients with ACL reconstruction complained of more knee pain than those without reconstruction and patients with major meniscal tear had more knee pain than those with intact menisci. Patients with radiographic tibiofemoral OA scored lower in all subscales of KOOS as compared to those without OA. The authors concluded that in patients willing to moderate their activity level, initial treatment without ACL reconstruction should be considered because favourable outcome in terms of knee function, symptoms and radiographic OA can be obtained in the long term with nonoperative treatment.

The study by Ajuied *et al*^19^ showed a higher risk of OA in patient treated conservatively as compared to those treated surgically while the study by Neuman *et al*^18^ showed that the patients treated by reconstruction of the ligament had a higher prevalence of OA. The study by Porat *et al*^20^ on the other hand showed that there was no difference in the prevalence between the two groups. These differences in different studies may be due to inconsistences in the acquisition and interpretation of radiographs as well as inconsistences in definition of OA and variation of the cohort of patients studied

The level 1 high quality study by Frobell *et al*^9^ has shown that there was no difference in the prevalence of OA in patients treated with rehabilitation, early ACL reconstruction or delayed ACL reconstruction. However in this study they found that the prevalence of patellofemoral OA was higher at 20% as compared to the tibiofemoral OA which was 12%. They also found that the prevalence of patellofemoral OA was higher in patients who had reconstruction with patellar tendon grafts as compared to hamstring tendon grafts. It is believed that shortening of the patella tendon after harvesting of the graft may increase the biomechanical loading of the patellofemoral joint leading to OA, as well as due to osteophyte formation that occurs due to bone remodelling after the graft is harvested. The conclusion from this study is that reconstruction of the ACL does not protect the knee from OA.

## Conclusion

The rationale for surgical treatment of an ACL tear was that the ACL is vital for knee function and that in the long term ACL deficiency will lead to more injuries of the meniscus and more degeneration of the joint. This belief was prevalent because the natural history of an ACL deficient knee was not known and the ultimate outcome of reconstruction of the ACL was also not known -. However over the past few years well conducted prospective studies have elucidated the natural history of conservatively treated knees with a tear of the ACL as well as the outcome of knees treated with reconstruction of the ACL.

We now can conclude that in the mid-term (5 years) there is no difference in the meniscal surgeries rates, radiographic osteoarthritis and all functional scores in young active patients treated with rehabilitation, early ACL reconstruction or delayed ACL reconstruction. We also can conclude that at 15 years (long term) the outcome after a tear of the ACL in term of knee function, symptoms and OA are good even with conservative treatment. Present day knowledge would dictate that the indication for surgery would be a tear of the ACL in elite sportsman who cannot alter their activity level and in patients who have recurrent giving way of the knee in spite of adequate rehabilitation.

About 50% of young active patients will need a delayed reconstruction of the ACL (level 1 study). However the cohort of patient in a general population who will need a delayed reconstruction of the ACL will only be about 23% (level 2 study) and these will be the type of patients that most orthopaedic surgeons in our country will treat.

These studies should produce a paradigm shift in the way we look at treatment of patients with injuries of the ACL. This would be in line with the requirement of critical enquiry that the concept of evidence based medicine dictates. With the present day scientific knowledge we cannot continue to justify surgical intervention by saying that the jury is out there as far as management of ACL injury is considered.

High level scientific evidence has not given solace to skeptics that most patients with an ACL injury will not need surgery^11^. Proponents who advocate ACL reconstruction will however find solace in a new study published recently in the Journal of Bone and Joint Surgery. = In this study by Mather *et al*^21^, the authors claim that early ACL reconstruction was less costly and more effective from the financial point of view then rehabilitation in the short to medium term and in the long term the life-time cost to society is lower following early ACL reconstruction. The authors however do admit that the study was based on several assumptions which were not backed by credible scientific evidence. The investigations in this study were carried out by a health consultancy firm. Some of the authors of the study had conflict of interest in the form of financial relationship with biomedical firms (Smith & Nephew and DonJoy Orthopaedics) which could be perceived to influence what has been published^22^.

Finally back to Mr Smith. Hopefully Mr Smith can now make an informed decision whether his doctor knows best.
